# Sensory and motor secondary symptoms as indicators of brain vulnerability

**DOI:** 10.1186/1866-1955-5-26

**Published:** 2013-09-24

**Authors:** Nava Levit-Binnun, Michael Davidovitch, Yulia Golland

**Affiliations:** 1 Interdisciplinary Center (IDC), Sagol Unit for Applied Neuroscience, School of Psychology, POB 167, Herzliya 46150, Israel; 2 Tel Aviv Maccabi Healthcare Services, 27 Hamered St., Tel Aviv 68125, Israel

**Keywords:** Motor, Network neuroscience, Networks, Resilience, Secondary symptoms, Sensory, Vulnerability

## Abstract

In addition to the primary symptoms that distinguish one disorder from the next, clinicians have identified, yet largely overlooked, another set of symptoms that appear across many disorders, termed *secondary symptoms*. In the emerging era of systems neuroscience, which highlights that many disorders share common deficits in global network features, the nonspecific nature of secondary symptoms should attract attention. Herein we provide a scholarly review of the literature on a subset of secondary symptoms––sensory and motor. We demonstrate that their pattern of appearance––across a wide range of psychopathologies, much before the full-blown disorder appears, and in healthy individuals who display a variety of negative symptoms––resembles the pattern of appearance of network abnormalities. We propose that sensory and motor secondary symptoms can be important indicators of underlying network aberrations and thus of vulnerable brain states putting individuals at risk for psychopathology following extreme circumstances.

## Introduction

In the clinical literature, disorders are characterized mainly by their distinguishing symptoms and behaviors, often referred to as the *primary symptoms*. For example, autism spectrum disorder (ASD) is defined by difficulties in communication and restricted behavior, attention-deficit/hyperactivity disorder (ADHD) by attention deficits and schizophrenia by reality distortion. In addition to these primary symptoms that distinguish one disorder from the next, there is another set of symptoms that appear across many disorders, termed *secondary symptoms* or *nonspecific signs*. *Secondary symptoms* refers to subtle impairments in motor coordination, complex motor sequencing, sensory integration and regulation, sleep and feeding difficulties, as well as difficulties in self-regulation [1,2]. Neurologists believe that secondary symptoms reflect nonlocalizable disturbances in connections between subcortical and cortical regions or among cortical regions [2]. Numerous publications have described the existence of these secondary symptoms in association with a variety of disorders. Yet, intriguingly, this body of work has been largely overlooked [3,4], perhaps because of the nonspecific nature of these “low-level” symptoms and the difficulty in relating them to the specific high-level impairments of the different disorders.

Although signs that appear across disorders may not be informative for understanding specific disorders, they may be highly relevant to understanding general characteristics of the brain in psychopathological states [4]. Indeed, the recent emergence of systems neuroscience, a promising field that calls for a paradigm shift in the way we view healthy and psychopathological brain states, suggests that invariant characteristics observed across psychopathologies may provide important information. Unlike traditional approaches that have focused on uniquely distinguishing one disorder from the next, there is an attempt in systems neuroscience to unify a wide range of psychopathologies and describe their common characteristics. That is, this field highlights that many disorders share common deficits in global network features [5]. The main focus of systems neuroscience lies in characterizing the abnormal brain architecture that underlies many disorders and describing the resulting consequences for the dynamic function of the brain network and its ability to maintain functional homeostasis. Indeed, one of the major consequences of the abnor-malities in brain architecture, observed in many disorders, is the loss of robustness of the brain network to perturbations. This suggests that brains with abnormalities in brain architecture are more vulnerable and that unfavorable circumstances (for example, psychological life stressors, chemical stressors, brain hemorrhages or aberrations in anatomical brain structures during development or neurodegeneration) can cause them to deviate from homeostasis.

Herein we propose an integrative framework in which secondary symptoms represent the behavioral manifestations of the brain’s abnormal architecture and are thus indicators of network vulnerability. To that end, we provide a systematic review of sensory and motor secondary symptoms and show that although secondary symptoms and network aberrations come from different levels of description—one at the behavioral level and the other at the network level—they display similar patterns of occurrence. That is, sensory and motor symptoms and network aberrations not only occur across a wide range of psychopathologies but also are detectable much before the full-blown disorder appears, in both at-risk populations and in healthy individuals who display a variety of negative symptoms. We then provide theoretical and empirical support that the behavioral level and the network level may be related and propose that secondary symptoms can be important indicators of vulnerable brain states at risk to psychopathology. In other words, the appearance of secondary symptoms in an individual may indicate that his brain is in a less resilient state, and thus he has a higher probability of developing psychopathology following unfavorable circumstances. The novel integrative approach used here yields practical clinical benefits and advances our understanding of the developmental paths of psychopathologies.

## Review

In this section, we review reports of sensory and motor secondary symptoms from a wide range of disciplines. We define sensory and motor symptoms broadly and focus on indications of abnormalities and difficulties observed in these processes. The primary purpose of this section is to reveal an underlying pattern of occurrence of these abnormalities in relation to psychopathology. Therefore, we investigated the occurrence of sensory and motor abnormalities during active states of psychopathology. In addition, we examined their occurrence before the psychopathology is evident, when there is a propensity to develop a psychopathology, and even in healthy, undiagnosed individuals at no apparent risk for psychopathology but with more negative symptoms than those found in the average healthy population.

## Methods

We conducted a systematic review of the literature on studies that linked signs of sensory and motor abnormalities to a wide range of developmental, psychiatric and/or affective and neurodegenerative psychopathologies. We used search terms that are variations of words related to sensory and motor abnormalities (see Table 1). Since neurologists often term the co-occurrence of sensory and motor abnormalities *neurological soft signs* (NSSs) [2], we also included this term in our search. The search was conducted in the following databases: APA PsychNET (http://psycnet.apa.org/), MEDLINE, Academic Search Complete  (http://www.ebscohost.com/academic/acade-mic-search-complete), Psychology and Behavioral Sciences Collection (http://www.ebscohost.com/academic/psychology-behavioral-sciences-collection),  SocINDEX (http://www.ebscohost.com/academic/socindex-with-full-text) and Education Resource Information Center (ERIC; http://eric.ed.gov/). The psychopathologies included in this search were autism, ADHD, dyslexia, learning disabilities, language disorders, schizophrenia, anxiety, depression, posttraumatic stress disorder (PTSD), obsessive-compulsive disorder (OCD), bipolar affective disorder, borderline personality disorder, Alzheimer disease (AD) and traumatic brain injury. We also searched for words related to healthy aging. In order to account for differences in definitions across disciplines (for example, schizophrenia can be characterized as a psychiatric disorder or a developmental one [6]), we included general search terms such as “psychiatric,” “developmental” or “affective.” The specific search terms used in this review, as well as exclusion criteria, are displayed in Table 1.

**Table 1:** **Search terms and exclusion criteria**^**a**^

**Category**	**Search terms**	**Exclusion criteria**
Motor	(motor OR movement OR motion OR “fine motor” OR “gross motor”) AND (abnormalities OR impairment OR difficulties OR dysfunction OR dysfunctions OR function OR disturbance OR disturbances OR deficient OR deficiency OR performance OR ability OR coordination OR skills OR profile OR behavior OR behaviour OR development OR synchrony)	In general, we searched for evidence indicating general abnormalities in motor performance. In cases in which little research had been performed on motor aspects of a certain disorder, however, we included studies that focused on more narrow motor tasks, such as finger-tapping. In general, however, we excluded studies that focused exclusively on interaction of motor task and cognitive load, handwriting abilities, visuomotor integration, motor imagery, physical fitness, representation of action, mirror neurons, visuospatial guidance of movement during imitation, motor imagery, motor speech impairments, movement planning, repetitive motor behavior, motor restlessness, dyskinesia and gait and/or posture problems.
Sensory	(sensory OR tactile OR auditory OR somatosensory OR sensorimotor OR balance OR vestibular OR sensation OR “sensory-motor”) AND (defensiveness OR modulation OR regulation OR processing OR sensitivity OR abnormalities OR responsivity OR hyperreactivity OR hyporeactivity OR “hyper-reactivity” OR “hypo-reactivity” OR impairment OR filtering OR difficulties OR dysfunction OR dysfunctions OR hypersensitivity OR hyposensitivity OR disturbance OR disturbances OR performance OR filtering OR intolerance)	We excluded animal studies, genetic studies and all studies that investigated sensory abnormalities in the brain via physiological measures (for example, sensory evoked potentials, fMRI studies and startle responses). In addition, we excluded studies dealing with reactivity to pain, sensory impairments (such as impairment in seeing or hearing), eye movements, motion or visuospatial perception, visual acuity, audiovisual speech, sensory memory, timing of sensory processes, sensory gating and speech. In addition, studies that looked at the temperamental aspect of “sensory seeking” were excluded.
Neurological soft signs	“neurological abnormalities” OR “neurological soft signs” OR “subtle neurologic compromise” OR “neurological examination abnormalities” OR “neurological signs” OR “soft signs” OR “neurological examination abnormalities” OR “neuropsychological”	Studies that focused on neurological deficits observable as cognitive and/or memory abnormalities were excluded. Only those that related to sensory and motor abnormalities were included.
Sleep	(sleep OR sleepiness OR insomnia OR sleep-related) AND (deprivation OR excessive OR symptoms OR regulation OR continuity OR continuity changes OR complaints OR disturbance OR disturbances OR deficits OR deficit OR disruption OR disruptions OR problems OR evaluation OR patterns)	Studies that focused exclusively on a certain sleep parameter (for example, REM activity, NREM activity, sleep spindle activity, slow-wave activity and K-complex activity) were excluded. In addition, we excluded studies focused on sleep difficulties resulting from sleep apnea or other breathing difficulties.
Autism spectrum disorder	autism OR ASD OR “autism spectrum” OR “autism spectrum disorder” OR “autistic spectrum” OR “autistic spectrum disorder” OR Asperger OR Asperger’s OR alexithymia	
Attention-deficit/hyperactivity disorder	ADHD OR “attention deficit hyperactivity disorder” OR “attention-deficit/hyperactivity disorder” OR “attention deficit disorder” OR “attention deficit disorder with hyperactivity” OR “deficit disorder attention” OR “attention difficulties” OR “hyperkinetic syndrome” OR “hyperkinetic disorder” OR “hyperactive child syndrome”	
Dyslexia and learning disabilities	dyslexia OR “learning disabilities” OR “learning deficits”	Studies that focused on higher-order visual and auditory processing abilities (such as speech discrimination) were excluded.
Language impairment	“specific language impairment” OR “speech delay” OR SLI	
Anxiety	anxiety OR anxious OR emotional behavior OR phobia OR phobias OR avoidant	
Posttraumatic stress disorder	PTSD OR “posttraumatic stress disorder” OR “post-traumatic stress disorder” OR “post traumatic stress disorder”	
Depression	depression OR depressive	We excluded studies of depression associated with another difficulty, such as migraine, menopause, cancer and chemotherapy. Postpartum depression, perinatal depression and parental depression were also excluded.
Bipolar affective disorder	“bipolar affective disorder” OR “bipolar disorder”	
Borderline personality disorder	borderline OR “borderline personality disorder”	
Obsessive-compulsive disorder	“obsessive compulsive disorder” OR OCD OR “repetitive behavior” OR “repetitive behaviors”	
Schizophrenia and psychosis	schizophrenia OR schizophrenic OR schizotypal OR psychosis	
Alzheimer disease	“Alzheimer disorder” OR Alzheimer OR Alzheimer’s OR dementia	
Traumatic brain injury	“traumatic brain injury: OR “traumatic brain injuries” OR TBI	
Normal aging	aging OR older people OR “older adults” OR “elder people” OR “elder adults” OR elderly	
General search terms	“psychiatric disorders” OR “psychiatric disorder” OR “psychotic disorders” OR “psychotic symptoms”	With regard to the “high-risk” search, we included only studies that dealt with risk for one of the disorders included in the review.
	“degenerative disorders” OR “neurological disorders	
	“affective disorders” OR “social-emotional behavior” OR “mood disorder” OR “mood disorders” OR mood OR “emotional disorders” OR “psychological distress” “high-risk” OR at-risk OR “at risk” OR “high risk”	

^a^ADHD, attention-deficit/hyperactivity disorder; ASD, autism spectrum disorder; fMRI, functional magnetic resonance imaging; NREM, non–rapid eye movement; OCD, obsessive-compulsive disorder; PTSD, posttraumatic stress disorder; REM, rapid eye movement; TBI, traumatic brain injury.

### Criteria for exclusion

Only reports that compared the appearance of sensory and/or motor abnormalities observed in individuals with a psychopathology with that of healthy human controls were accepted into the review. Comorbidities (for example, ADHD together with developmental coordination disorder) were excluded. As mentioned, our goal was to present a pattern of appearance of secondary symptoms as reported by researchers and clinicians. Thus, we did not include reviews and meta-analyses. We also did not distinguish between research methodologies (questionnaires, retrospective studies, prospective studies and objective tests), as long as they presented quantifiable data that could determine whether or not a difference was observed between the psychopathology tested and healthy controls. Thus, single-case studies, qualitative studies and intervention studies were not included. In addition, most of the vast neurophysiological literature fell outside the scope of this review (for example, anatomical, sensory event–related potentials, imaging and electrophysiological studies). Additional exclusion criteria are presented in Table 1.

### Division of included studies into four categories

The studies that were included in the review were divided into four categories: (1) irregularities across disorders: that is, studies demonstrating that sensory and/or motor abnormalities appear in a full-blown psychopathology (for example, sensory abnormalities in individuals with autism); (2) irregularities before primary symptoms: studies demonstrating that sensory and/or motor abnormalities are evident before the full-blown symptoms of a psychopathology appear (for example, a retrospective study showing that children with ADHD displayed motor abnormalities as infants or a prospective study of individuals at risk for schizophrenia who later developed the disorder) or studies of populations considered at risk for a disorder (for example, findings of sensory abnormalities in siblings of patients with autism); (3) irregularities in healthy individuals: studies which assessed rates of sensory and/or motor abnormalities in healthy populations not considered at risk for psychopathology (for example, healthy college students); (4) no difference: studies that indicated no sign of sensory and/or motor abnormalities in relation to psychopathology (for example, no difference between individuals with autism and healthy controls in sensory abnormalities). The fourth category was included to enable a balanced picture.

## Results

Our search revealed more than 330 reports that mentioned sensory or motor irregularities or their combination (that is, NSSs) in relation to psychopathology or symptoms of psychopathology. Dividing them into our four categories revealed a clear pattern of appearance.

### Irregularities across disorders

Two hundred thirty-two reports mentioning sensory or motor irregularities in individuals with psychopathology were found (see Table 2 for details). Although there was variability in the number of published reports relating sensory and/or motor irregularities to specific disorders (ranging from three papers implicating secondary symptoms in PTSD and fifty-two implicating them in autism), we found mentions of sensory and/or motor irregularities for each of the search terms. Only 22 reports found no clear differences between the rate of sensory and/or motor signs in individuals with a psychopathological condition and healthy people (see right column in Table 2).

**Table 2:** **Sensory and/or motor irregularities appear across disorders**^**a**^

**Disorder**	**Sensory and/or motor irregularities appear more frequently in disorder than in healthy controls**	**Not different from healthy controls**
Autism	Sensory	Sensory
	Adamson *et al.*, 2006; Adrien *et al.*, 1987; Baranek, 1999; Baranek *et al.*, 2007; Baranek *et al.*, 2006; Baranek *et al.*, 2013; Ben-Sasson *et al.*, 2007; Blakemore *et al.*, 2006; Blanche *et al.*, 2012; Cascio *et al.*, 2008; Cheung and Siu, 2009; Crane *et al.*, 2009; Dickie *et al.*, 2009; Dunn *et al.*, 2002; Hilton *et al.*, 2010; Hochhauser and Engel-Yeger, 2010; Kern *et al.*, 2008; Kern *et al.*, 2007a; Kern *et al.*, 2007b; Kern *et al.*, 2006; Kern *et al.*, 2007c; Khalfa *et al.*, 2004; Kientz and Dunn, 1997; Kwakye *et al.*, 2011; Leekam *et al.*, 2007; O’Brien *et al.*, 2009; Reynolds *et al.*, 2011; Reynolds *et al.*, 2012; Rogers *et al.*, 2003; Siaperas *et al.*, 2012b; Tavassoli and Baron-Cohen, 2012b; Tomchek and Dunn, 2007; Watling *et al.*, 2001; Weimer *et al.*, 2001; Woodard *et al.*, 2012 [7-41]	Fuentes *et al.*, 2011; Güçlü *et al.*, 2007; Jones *et al.*, 2009; Tavassoli and Baron-Cohen, 2012 [42-45]
	Motor	Motor
	Dewrang and Sandberg, 2010; Esposito and Venuti, 2008; Freitag *et al.*, 2007; Gernsbacher *et al.*, 2008; Hilton *et al.*, 2007; Hilton *et al.*, 2012; Lopata *et al.*, 2007; Pan *et al.*, 2009; Papadopoulos *et al.*, 2012; Sahlander *et al.*, 2008; Siaperas *et al.*, 2012; Travers *et al.*, 2013; Whyatt and Craig, 2012 [34,46-57]	Ozonoff *et al.*, 2008 [58]
	NSS	
	De Jong *et al.*, 2011; Jansiewicz *et al.*, 2006; Jones and Prior, 1985; Tani *et al.*, 2006 [59-62]	
Language disorders	Sensory	
	Taal *et al.*, 2013 [63]	
	Motor	
	Chuang *et al.*, 2011; Finlay and McPhillips, 2013; Müürsepp *et al.*, 2009; Owen and McKinlay, 1997; Zelaznik and Goffman, 2010 [64-68]	
Dyslexia	Sensory	Sensory
	(Auditory visual processing deficits)	
	Fraser *et al.*, 2010; Georgiou *et al.*, 2012; Heiervang *et al.*, 2002; White *et al.*, 2006; Wright and Conlon, 2009 [69-73]	Polatajko, 1985; White *et al.*, 2006 [74,75]
	(Balance and tactile)	
	Brookes *et al.*, 2010; Cinelli and DePaepe, 1984; Getchell *et al.*, 2007; Kinnealey, 1989; Needle *et al.*, 2006; Nicolson and Fawcett, 1994 [76-81]	
	(Balance and tactile)	
	Bruininks and Bruininks, 1977; Cermak *et al.*, 1990; Durand, 2005; Getchell *et al.*, 2007; Haslum and Miles, 2007; Maloy and Sattler, 1979; McPhillips and Sheehy, 2004; Pieters *et al.*, 2012; Trauner *et al.*, 2000; Vuijk *et al.*, 2011; Westendorp *et al.*, 2011 [80,82-91]	
ADHD	Sensory	Sensory
	Bröring *et al.*, 2008; Cheung and Siu, 2009; Dunn and Bennett, 2002; Engel-Yeger and Ziv-On, 2011; Lufi and Tzischinsky, 2012; Miller *et al.*, 2012; Romanos *et al.*, 2008 [40,92-97]	Gomez and Condon, 1999; Schlee *et al.*, 2012 [98,99]
	Motor	Motor
	Flapper *et al.*, 2006; Chan *et al.*, 2010; Fliers *et al.*, 2009; Fliers *et al.*, 2010; Goulardins *et al.*, 2013; Harvey *et al.*, 2007; Karatekin *et al.*, 2003; Klimkeit *et al.*, 2004; Klotz *et al.*, 2012; Meyer and Sagvolden, 2006; Okuda *et al.*, 2011; Pan *et al.*, 2009; Piek *et al.*, 1999; Rommelse *et al.*, 2007; Slaats-Willemse *et al.*, 2005 [47,100-113]	Kooistra *et al.*, 2005; Lee *et al.*, 2013; Polderman *et al.*, 2011 [114-116]
	NSS	
	Chan *et al.*, 2010; Dickstein *et al.*, 2005; Ferrin and Vance, 2012 [102,117,118]	
Anxiety	Motor	Motor
	Ekornås *et al.*, 2010; Kristensen and Torgersen, 2007; Skirbekk *et al.*, 2012 [119-121]	Jacob *et al.*, 2009 [122]
	Sensory	
	Erez *et al.*, 2004; Farrow and Coulthard, 2012; Hofmann and Bitran, 2007 [123-125]	
	NSS	
	Hollander *et al.*, 1996 [126]	
Posttraumatic stress disorder	NSS	NSS
	Gurvits *et al.*, 2000; Gurvits *et al.*, 1993; Gurvits *et al.*, 2006 [127-129]	Gurvits *et al.*, 2002 [130]
Depression	Motor	NSS
	Günther *et al.*, 1988; Lohr *et al.*, 2013; Schwartz *et al.*, 1990 [131-133]	Zhao *et al.*, 2013 [134]
	NSS	
	Boks *et al.*, 2004; Manschreck and Ames, 1984 [135,136]	
Bipolar affective disorders	Motor	Sensory
	Dickstein *et al.*, 2005; Lohr and Caligiuri, 2006 [117,137]	Swiecicki *et al.*, 2009 [138]
	NSS	
	Negash *et al.*, 2004; Zhao *et al.*, 2013 [134,139]	
Borderline personality disorder	Sensory	Sensory
	Brown *et al.*, 2009; Rosenthal *et al.*, 2011 [140,141]	Pavony and Lenzenweger, 2013 [142]
	Motor	
	Swirsky-Sacchetti *et al.*, 1993 [143]	
	NSS	
	De la Fuente *et al.*, 2011; De la Fuente *et al.*, 2006; Gardner *et al.*, 1987 [144-146]	
Schizophrenia	Sensory	Sensory
	Brown *et al.*, 2002; Cheng *et al.*, 2012; Colbert *et al.*, 1959; Emmerich and Levine, 1970; Ghadirian and Butter, 1978; Kent *et al.*, 2012; Kiss *et al.*, 2010; Levine and Whitney, 1970; Myers *et al.*, 1973; Ramage *et al.*, 2012 [147-156]	Levy *et al.*, 1978 [157]
	Motor	Motor
	Günther *et al.*, 1986; Midorikawa *et al.*, 2008; Sullivan *et al.*, 1994; Tabarés-Seisdedos *et al.*, 2003 [158-161]	Martin *et al.*, 1995 [162]
	NSS	
	Aksoy-Poyraz *et al.*, 2011; Aydemir *et al.*, 2005; Boks *et al.*, 2004; Chan and Chen, 2007; Chen *et al.*, 2000; Compton *et al.*, 2007; Flyckt *et al.*, 1999; Ismail *et al.*, 1998; Jaafari *et al.*, 2011; Le Seac’h *et al.*, 2012; Manschreck and Ames, 1984; Mechri *et al.*, 2009; Mohr *et al.*, 1996; Nucifora and Calandra, 1999; Sevincok *et al.*, 2004; Shibre *et al.*, 2002; Venkatasubramanian *et al.*, 2003; Walker and Green, 1982; Yazici *et al.*, 2002; Zhao *et al.*, 2013 [134-136,163-179]	
Obsessive-compulsive disorder	Motor	NSS:
	Bloch *et al.*, 2011 [180]	Jaafari *et al.*, 2011 [172]
	Sensory	
	Dar *et al.*, 2012; Rieke and Anderson, 2009; Segalàs *et al.*, 2011 [181-183]	
	NSS	
	Bolton *et al.*, 1998; Hollander *et al.*, 1990; Karadag *et al.*, 2011; Mataix-Cols *et al.*, 2003; Peng *et al.*, 2012 [184-188]	
Alzheimer disease	Sensory	
	Gallacher *et al.*, 2012; Kato-Narita *et al.*, 2011; Leandri *et al.*, 2009; Pettersson *et al.*, 2002; Pettersson *et al.*, 2005; Rolland *et al.*, 2009; Suttanon *et al.*, 2012; Waite *et al.*, 2000 [189-196]	
	Motor	
	Kluger *et al.*, 1997; Kluger *et al.*, 1997; Oakley *et al.*, 2003; Ott *et al.*, 1995; Pettersson *et al.*, 2005 [193,197-200]	
	NSS	
	Lam *et al.*, 2005; Seidl *et al.*, 2009 [201,202]	
Traumatic brain injury	Sensory	
	Gagnon *et al.*, 1998; Galvin *et al.*, 2009; Katz-Leurer *et al.*, 2008; Kuhtz-Buschbeck *et al.*, 2003 [203-206]	
	Motor	
	Gagnon *et al.*, 1998 [203]	
Normal aging	Sensory	Sensory
	Cole, 1991; Desrosiers *et al.*, 1996; Fukunaga *et al.*, 2005; Li *et al.*, 2004; Petrosino and Fucci, 1989; Pohl *et al.*, 2003; Ranganathan *et al.*, 2001; Schumm *et al.*, 2009; Shimokata and Kuzuyam, 1995; Stevens, 1992; Stevens and Cruz, 1996; Zhang *et al.*, 2011 [207-218]	Bartoshuk *et al.*, 1986; Elsner, 2001 [219,220]
	Motor	Motor
	Ashendorf *et al.*, 2009; Bennett *et al.*, 2012; Kauranen and Vanharanta, 1996; Smith *et al.*, 1999; Vernazza-Martin *et al.*, 2008 [221-225]	Kleinman, 1982; Santos *et al.*, 2007 [226,227]
	NSS	
	Chan *et al.*, 2011 [228]	

^a^ADHD, attention-deficit/hyperactivity disorder; NSS, neurological soft sign.

#### ***Irregularities before primary symptoms***

Our search revealed 60 retrospective, prospective and sibling and/or family studies indicating that abnormalities in sensory and motor processes are evident before psychopathology appears and before the full-blown symptoms are visible (see Table 3 for details). Such findings suggest that these irregularities not only are correlated with the disorders but may have a predictive nature as well [3,229,230].

**Table 3:** **Sensory and/or motor irregularities appear long before the full-blown symptoms**^**a**^

**Disorders**		**At-risk populations**	**Prospective and retrospective studies**
Developmental disorders	Autism	Bryson *et al.*, 2007; Mulligan and White, 2012, Bhat *et al.*, 2012; Bryson *et al.*, 2007; Landa and Garrett-Mayer, 2006; Landa *et al.*, 2012; Ozonoff *et al.*, 2008 [58,231-235]	Baranek, 1999; Landa *et al.*, 2013 [9,236]
	ADHD	Fliers *et al.*, 2009; Rommelse *et al.*, 2007 [100,112]	Karatekin *et al.*, 2003; Kroes *et al.*, 2002 [107,237]
	Dyslexia	Viholainen *et al.*, 2002; Viholainen *et al.*, 2006 [238,239]	Cannon *et al.*, 1999; Clarke *et al.*, 2011; Murray *et al.*, 2006; Rosso *et al.*, 2000; Schiffman *et al.*, 2009; Walker and Lewine, 1990; Watkins *et al.*, 1988 [240-246]
Psychiatric disorders	Schizophrenia	Aksoy-Poyraz *et al.*, 2011; Blanchard *et al.*, 2010; Chan *et al.*, 2010; Chang and Lenzenweger, 2001, 2005; Chen *et al.*, 2000; Compton *et al.*, 2007; Egan *et al.*, 2001; Fish, 1976; Fish and Dixon, 1978, Fish and Hagin, 1973; Gschwandtner *et al.*, 2006; Ismail *et al.*, 1998; Koning *et al.*, 2011; Marcus *et al.*, 1993; Mechri *et al.*, 2009; Mechri *et al.*, 2010; Mittal *et al.*, 2007; Mittal *et al.*, 2008; Neumann and Walker, 2003; Picchioni *et al.*, 2006; Prasad *et al.*, 2009; Rieder and Nichols, 1979; Walker *et al.*, 1999; Yazici *et al.*, 2002 [247][248-250][251-253][254][163-166,171,247-265]	
Affective disorders	Depression	Harjan, 1989 [266]	Pine *et al.*, 1993; Shaffer *et al.*, 1985 [267,268]
	Anxiety	Turner *et al.*, 2005 [269]	Shaffer *et al.*, 1985; Sigurdsson *et al.*, 2002 [268,270]
	Bipolar		Sigurdsson *et al.*, 1999) [271]
	OCD	Peng *et al.*, 2012 [184]	Grisham *et al.*, 2011 [272]
	PTSD	Gurvits *et al.*, 2006 [129]	
Degenerative disorders	Aging		Inzitari *et al.*, 2006 [273]
	Alzheimer disease	Aggarwal *et al.*, 2006; Rolland *et al.*, 2009; Scarmeas *et al.*, 2005 [194,274,275]	

^a^ADHD, attention-deficit/hyperactivity disorder; NSS, neurological soft sign; OCD, obsessive-compulsive disorder; PTSD, posttraumatic stress disorder.

For example, various sensory and motor difficulties precede communication delay and autism symptoms in most infants who later develop ASD [9,58,232] or who are at risk for developing autism [231]. In toddlers at risk for familial dyslexia, researchers found a relationship between motor development at infanthood and the level of language skills at toddlerhood [238]. In a prospective study following 401 toddlers, motor performance was found to be predictive of ADHD [237]. In another study comprising 275 children with ADHD, their siblings and controls, the unaffected siblings displayed intermediate levels of motor problems between the ADHD-affected children and the control subjects [100].

Quite a few studies have reported the existence of sensory and motor signs much sooner than schizophrenia symptoms were evident. For example, a few prospective studies [240-242] found that children who developed schizophrenia as adults took longer to achieve motor milestones and scored significantly worse than controls on motor coordination tasks. Significantly more NSSs were found in children and relatives with a higher genetic risk for schizophrenia [248,276] and in those who later became adult schizophrenics [242]. Moreover, relatives of patients with schizophrenia exhibited levels of NSSs that were intermediate between patients with the full-blown disorder and healthy controls [163-166].

Early motor signs were also predictive of later mood-related psychopathologies. For example, in a large cohort study of 6,850 children, boys with poor motor skills at age 7 years had more than three times the odds of having maternally rated anxiety at the ages of 11 and 16 years [270]. Another study found that both OCD patients and their unaffected first-degree relatives displayed more motor coordination signs than healthy controls [184]. In several prospective epidemiological studies, an association was found between NSSs and the development of anxiety, depression and obsessive-compulsive symptoms over time [267,268]. Interestingly, recent studies on twins have found that NSSs represent vulnerability to PTSD. Not only did combat veterans with PTSD have significantly more NSSs than combat veterans without PTSD, but their unexposed co-twins had significantly more NSSs than the unexposed co-twins of the veterans without PTSD [129,277].

### Irregularities in healthy individuals

Although researchers rarely examine signs of psychopathology in healthy individuals, our search succeeded in revealing 17 studies that reported the occurrence of sensory and motor abnormalities in healthy individuals. Interestingly, in all of these reports, negative mental symptoms related to psychopathology (for example, higher anxiety and depression levels) were found to correlate with higher rates of sensory and/or motor abnormalities (see Table 4). That healthy individuals with more sensory and motor abnormalities than average also tend to have more negative mental health symptoms suggests that these abnormalities are not the consequence of psychopathology, but rather a characteristic of an underlying vulnerability to it.

**Table 4:** Sensory and/or motor irregularities are associated with negative symptoms in healthy individuals

**Signs of psychopathology**	**References**
Anxiety and depression symptoms	Bart, 2009; Kogan, 2008; Lane, 2010; Kinnealey, 1999; Engel-Yeger, 2011; Liss, 2008; Liss, 2005; Goldsmith, 2006; Piek, 2008; Pearsall-Jones, 2011; Piek, 2010; Kinnealey, 1999 [278-288]
Alexithymia and autistic characteristics	Liss, 2008; Robertson, 2013 [287,289]
Social phobia symptoms	Neal, 2002 [290]
Avoidant and borderline personality traits	Meyer, 2005; Meyer, 2000 [291,292]
Psychiatric symptoms	Ben-Sasson, 2009; Gouze, 2009; Stansfeld, 1985 [293-295]

For example, healthy individuals with extreme sensory reactivity are more likely to display signs of anxiety and depression [278-281,287,296]. Individuals with extreme sensory reactivity patterns also display higher signs of alexithymia and autistic characteristics [287,289], social phobia [290] and avoidant and borderline personality traits [291,292]. Preschoolers and school-aged children with sensory regulation dysfunction (for example, sensory overresponsivity) were more likely to display early and co-occurring internalizing and externalizing behaviors, lower levels of concurrent adaptive social behaviors and more psychiatric symptoms [293,294]. Similarly, preschoolers with motor coordination difficulties exhibited negative emotional symptoms, such as signs of depression and anxiety [282,288]. This was also shown in a twin study, which found significantly higher levels of anxious and depressive symptomatology in twins with motor abnormalities compared with control twins with no motor difficulties. Moreover, in twins discordant for motor abnormalities, the twin with motor difficulties exhibited more anxious and depressive signs than the co-twin without such difficulties [283].

### Summary

Our systematic review revealed a clear pattern of the appearance of sensory and motor irregularities across developmental, affective and/or psychiatric and degenerative brain psychopathologies. These irregularities not only appear together with the full-blown disorder but also are apparent much before its primary signs, even in individuals who are considered healthy. Although clinicians have long noted the nonspecific nature of sensory and motor signs in relation to many disorders, to the best of our knowledge, this review is the first scholarly demonstration of their pattern of appearance. Notably, other irregularities included within the group of secondary symptoms––such as self-regulation and eating and sleeping difficulties––have also been associated with a wide range of disorders. Although a systematic review of these symptoms is still lacking, they seem to follow a similar pattern (see [4] for examples). Taken together, the facts that secondary symptoms are widespread and that they are even associated with the extreme end of the healthy subclinical spectrum indicate a need for a reexamination of their possible contribution to understanding the pathways to psychopathology. Next, we suggest that the nonspecific nature of these secondary symptoms, which may have led to their being overlooked by clinicians and researchers, may actually be an advantage in the eyes of the emerging field of systems neuroscience.

### Explaining the pattern of occurrence of sensory and motor secondary symptoms within a systems neuroscience framework

#### ***Outlining the systems neuroscience framework***

Driven by methodological advances concerned with the study and analysis of complex networks in the brain, the exciting new field of systems neuroscience offers a novel perspective on ways to examine the brain, both in health and in psychopathology [5,297]. Systems neuroscientists use graph theoretical tools to assess various network metrics^1^[1] from a large body of functional and structural connectivity data obtained from diffusion tensor imaging (DTI), functional magnetic resonance imaging (fMRI), magnetoencephalography (MEG) and electroencephalography (EEG) studies. The network metrics obtained from these data allow for the examination of normal and psychopathological brain states within a framework of global network structures (see, [5], for example), thereby indicating the efficiency of information transfer across the whole network and the ability of large-scale networks within the brain to switch between engaged and disengaged modes of function.

For example, converging evidence indicates that healthy brains are characterized by small-world architecture [298,299], which describes a configuration in which most nodes are not neighbors, but can be reached from every other node by a small number of steps. Such an architecture enables a balance between local and global structural characteristics and thus an optimal balance between segregation and integration, which is essential for high synchronicity and fast information transmission in a complex network [300].

Furthermore, it is believed that the construction of brain networks (as well as most biological networks) in small-world architecture may reflect an evolutionary advantage. One possibility is that small-world networks are more robust to perturbations than other network architectures [301,302]. This robustness is supported by global network mechanisms [302] that limit the effects of potentially disruptive perturbations [301-304]. For example, the high modularity and degeneracy characteristics of small-world networks enables the insulation of subsystems that are functionally connected to each other from spreading perturbations (due to high modularity) and supports functional homeostasis even in the face of large structural variation in structure (due to the existence of many degenerate pathways) [305]. The inhibitory connections spread throughout the network play an important role in maintaining dynamic balance, whereas self-organizing regulatory mechanisms support functional homeostasis [306]. All of these network mechanisms maintain the remarkable capacity of the brain to withstand a wide range of perturbations in the course of injury and disease [302]. Thus, abnormalities in network metrics can result in general network failure, which affects network robustness as well as efficient information transfer, and can cause deficits in the access, engagement and disengagement of large-scale networks supporting cognition and behavior [302,307].

#### ***Aberrations in network metrics follow a similar pattern of occurrence***

Using network analytical methods, researchers who have conducted empirical and theoretical studies have found that aberrations in various network metrics are a telltale sign of significant global deficits in brain organization [5] and that psychopathology is related to dysfunctional brain organization [302,308]. Convergent anatomical and functional evidence from a wide range of methodologies and from many different psychopathological states indicates that the disruption of global network connectivity, as indicated by abnormalities in various network metrics, is associated with disturbances in cognition and behavior as well as with signs of psychopathology [309-312].

A range of neurodevelopmental disorders have already been described in terms of aberrant network measures. In an EEG study comparing children with autism to healthy controls, children with autism showed a loss of small-world architecture, characterized by a significantly increased path length and reduced clustering [313,314]. Similar findings were also demonstrated for high-functioning adults with autism and for those with Asperger syndrome [315,316]. Another neurodevelopmental disorder that has been studied using a network approach is ADHD. Using resting-state fMRI data, several studies have revealed alterations in small-world architecture, characterized by increased local network efficiencies and decreases in global network efficiencies, in both children and adults with ADHD [317-319].

Schizophrenia, a psychiatric disorder that is considered to be neurodevelopmental in origin [6], has also been studied extensively using network analytical tools [320-322]. For example, a comparison of structural data from schizophrenia patients and healthy controls indicated a loss of small-world architecture as measured by a significantly lower clustering coefficient, longer characteristic path length and dysfunctional central hubs [320,323].

Abnormalities in network metrics were also reported for affective disorders as well as for neurodegenerative disorders and aging. For example, aberrant small-world architecture was found for drug-naïve, first-episode major depression patients [324] and for patients with OCD [325]. Abnormalities in network metrics were also reported when functional connectivity data from patients with AD [326-329], dementia [330] and traumatic brain injury [331] were compared to those from controls. Interestingly, aberrations in network metrics were also observed in healthy elders compared to healthy younger adults [332,333]. Taken together, these studies suggest that network aberrations are a general characteristic of psychopathologies without regard to the causes leading to the psychopathology.

Perhaps even more interesting is the fact that disruptions in network metrics seem to occur even before the full-blown disorder is evident [334-337]. This is most obvious in AD and schizophrenia, which have been studied extensively in this respect. For example, Yao *et al.*[334] measured network parameters in patients with AD, individuals with mild cognitive impairments (MCIs) and healthy age-matched controls. The longest absolute path length and the greatest clustering coefficient were found in patients with AD, which indicates that the small-world organization of the cortical network was the least optimal in AD. The small-world measures of the MCI network exhibited intermediate values between AD and the normal aging controls. Given that MCI is a transitional stage between normal aging and AD [334], these findings, as well as those reported in other studies [338-341], suggest that network aberrations are evident in at-risk individuals much before the full-blown AD symptoms appear [334].

With regard to schizophrenia, Dazzan *et al.*[342] obtained MRI data from 102 individuals considered to be at a very high risk for schizophrenia and followed them for one year. Those who developed schizophrenia or other forms of psychosis had more volumetric abnormalities in distributed brain areas at the time of the MRI scan than those who did not develop a form of psychosis. That risk for schizophrenia is associated with aberrant brain architecture is further supported by additional studies indicating pervasive brain anatomical abnormalities in individuals at high risk for schizophrenia [343,344], even as early as neonatal stages [345]. Shi *et al*. found that neonates at genetic risk for schizophrenia tended to have more aberrant metrics––such as globally lower efficiency and longer connection distance––than healthy controls, which indicates a less optimal small-world structures [345].

Further support that network aberrations predate the onset of a psychopathology is provided by studies demonstrating that healthy individuals with autistic traits [346] and healthy individuals with familial risk for ADHD [347] display more network abnormalities compared to healthy individuals with no risk for psychopathology. Dennis *et al*. [348] showed that structural brain networks of healthy individuals (measured with DTI) carrying one of the known autism risk genes (*CNTNAP2*) exhibited altered structural connectivity that was reflected in aberrant path length, small-world structure and global efficiency as compared to non-at-risk individuals.

To summarize, similarly to the case of the secondary symptoms reviewed above, network aberrations seem to accompany many brain pathologies and appear in at-risk individuals even before a psychopathology is diagnosed. Furthermore, they seem to be predictive in nature. As mentioned above, abnormalities in global network metrics are related to the loss of the network’s robustness and to an increase in vulnerability. In the next section, we suggest that secondary symptoms are linked to network aberrations and thus to brain vulnerability.

#### ***Secondary symptoms as indicators of network vulnerability***

Central to the framework developed herein is the observation that although sensory and/or motor secondary symptoms and network aberrations come from different levels of description—one at the behavioral level and the other at the network level—they display similar patterns of occurrence. They are both found across psychopathologies, before psychopathology emerges, and seem to have some predictive capability. Both theoretical and empirical evidence suggests that they may indeed be related.

On a theoretical level, the relation between secondary symptoms and network aberrations can be supported by a deeper understanding of the consequences of the loss of small-world architecture and abnormalities in network metrics. As mentioned above, small-world architecture enables greater robustness to perturbations, as well as more efficient information transfer and integration throughout the network. Moreover, the architecture of small-world networks gives rise to global network mechanisms that ensure the maintenance of dynamic balance. Thus, network failure leads not only to less robustness to perturbations but also to less efficient information transfer throughout the network, as well as to the disruption of mechanisms in charge of maintaining dynamic balance [302]. As a result, network failure should affect general network function, including the most basic input, output and regulation processes [302]. Thus, irregularities in sensory, motor and regulatory processes can be viewed as arising together with the loss of robustness due to network failure. In other words, the same network abnormalities that lead to loss of robustness can also lead to abnormalities in basic processes (that is, secondary symptoms).

Empirically, this proposed relation between secondary symptoms and network aberrations is supported by a recent set of experiments demonstrating that sensory and motor secondary symptoms are associated with structural brain irregularities. Dazzan *et al*. investigated NSSs in 43 healthy individuals using high-resolution MRI and voxel-based methods of image analysis [349]. They found that higher rates of NSSs were associated with a reduction in the size of the inferior frontal gyrus, the middle and superior temporal gyrus, and the anterior cingulate gyrus. Several other works have recently found a strong association between the occurrence of NSSs in patients with schizophrenia and structural abnormalities in subcortical brain morphology [350-354]; cerebello-thalamo-prefrontal networks [355]; the corpus callosum [356]; and prefrontal, temporal and cerebellar structures [356-358]. Signs of sensory integration deficits have been associated with volume reduction in the cerebral cortex, including the precentral, superior and middle temporal, and lingual gyri [354]. In a longitudinal study, Kong *et al*. [359] investigated the cerebral correlates of persisting NSSs in first-episode patients with schizophrenia. They found that patients with a greater number of persistent neurological signs not only showed a less favorable outcome after one year (although differences did not reach significance) but also had significantly more structural abnormalities, which indicated a larger number of progressive cerebral changes. Taken together, these observations suggest that sensory and/or motor abnormalities represent clinical indicators of perturbed connectivity that underlies psychopathology [349,353,354,360,361].

Thus, network theory suggests that loss of network robustness can arise together with irregularities in basic processes due to global network failure [302]. In addition, structural evidence suggests that sensory and/or motor abnormalities represent clinical indicators of abnormalities in brain architecture [349-361]. We thus suggest a novel integration between clinical observations and systems neuroscience that can explain the similarities between the patterns of occurrence of sensory and/or motor abnormalities and network aberrations. Within this framework, we propose that sensory and motor secondary symptoms reflect irregularities in basic processes arising because of network irregularities and are therefore indicators of vulnerable brain networks. In other words, they are a behavioral marker for a brain that is already in a psychopathological state or has the potential to develop a psychopathology under certain conditions.

The last part of our proposition suggests that prevalent appearance of secondary symptoms has a predictive nature. Thus, it can serve as a marker for individuals in whom psychopathology is not evident but has the potential to evolve. This is supported by many levels of evidence that we have referred to in this review, namely, the following: (1) that sensory and/or motor abnormalities appear in healthy individuals who are considered at risk for psychopathology (Table 3), (2) that sensory and/or motor abnormalities appear in healthy individuals not considered at risk for psychopathology but who display more negative symptoms than the average healthy population (Table 4), (3) that higher rates of NSSs in healthy people are associated with structural abnormalities [349] and (4) that healthy people at risk for psychopathology display network abnormalities.

## Discussion

In this review, we emphasize the underlying pattern of occurrence of abnormalities in sensory and motor processes that seem to appear during states of psychopathology, before the psychopathology is evident and when there is a propensity to develop a psychopathology. Furthermore, our review demonstrates that even in healthy, undiagnosed individuals at no apparent risk for psychopathology, deviations from the norm in basic processes are correlated with more negative symptoms than those found in the average healthy population.

In addition, we show that abnormalities in network metrics associated with psychopathology exhibit a pattern of occurrence similar to that of secondary symptoms. We offer a novel integration between clinical observations and systems neuroscience to try to gain an understanding of the meaning of this pattern. This synthesis yielded a proposition that abnormalities in sensory and motor processes can be indications of a vulnerable brain (that is, a brain that has a higher probability of developing psychopathology following extreme circumstances).

Importantly, herein we focus only on a subset of possible secondary symptoms (sensory and/or motor). Although a similar systematic review of other secondary symptoms, such as sleeping, eating and self-regulation abnormalities, is lacking, a nonsystematic review that has been performed suggests that their pattern of appearance in relation to psychopathology resembles that of the sensory and/or motor secondary symptoms [4]. This similarity in patterns suggests that there are other indicators of brain vulnerability in addition to the sensory and/or motor symptoms reviewed herein.

The framework outlined herein supports the emerging theoretical approach that calls for a paradigm shift in the way psychopathologies are viewed, diagnosed and treated [3-5]. According to this approach, the current trend—in which syndromes are compartmentalized and viewed as exclusive and separable from each other and in which emphasis is placed on finding early markers that will predict specific disorders—is misguided [3]. The fact that many symptoms are shared across psychopathologies [3,5] and that disorders often appear together suggests that many disorders (for example, ASD and ADHD) are actually representations of different aspects of the same underlying abnormality (for example, aberrations in global networks) [3,362]. Thus, symptoms that appear across disorders “should be considered markers for the (very likely) presence of a neurodevelopmental disorder that (very likely) will continue to cause symptoms long after their clinical surfacing” [3]. In accordance with this recent approach, our framework suggests that secondary symptoms, such as sensory and/or motor abnormalities, represent an underlying potential (due to network vulnerability) to develop psychopathology. The prevalence and severity of secondary symptoms may indicate the level of this vulnerability [363].

Whether this potential to develop psychopathology will be realized and how it will be expressed depend both on the “level of vulnerability” of the underlying network and on the existence, nature and timing of a trigger that can shift the system from healthy to psychopathological function. For example, a trigger can be the abnormal anatomical or synaptic changes that occur during powerful developmental or degenerative processes. Extreme circumstances can also serve as triggers (for example, stressful life adversities, chemical stressors or brain hemorrhages). Indeed, the interaction between developmental aberrations and external stressors is exemplified in schizophrenia. Growing evidence indicates that schizophrenia is a developmental disorder (see, for example, [6]). However, it usually erupts only during the stressful period of late adolescence and early adulthood, when young adults leave their parents’ home and become independent [364]. The integrative framework presented herein emphasizes the need to better understand the interaction between the prevalence and severity of secondary signs, as well as the timing and nature of perturbations that can trigger the development of a psychopathology.

Several important clinical implications, relating to both early detection and intervention, arise from this framework. This review gathers an extensive body of supportive evidence showing that the appearance of nonspecific secondary symptoms, as well as nonspecific metric aberrations, precedes the development of specific psychopathologies. Early detection of vulnerability may therefore contribute to efforts directed at finding early markers of psychopathology [3,4]. Further research is needed to directly assess the clinical usefulness of secondary symptoms as predictors of psychopathology.

Regarding implications for interventions, it is possible that, once secondary symptoms are detected, an intervention could reduce vulnerability and prevent or curtail the development of psychopathology. Interestingly, preliminary evidence suggests that interventions targeting basic processes associated with secondary symptoms (for example, sensory, motor or sleep) seem to alleviate vulnerability in preterm infants and in individuals already diagnosed with a psychopathology (see, for example, [365-370]). These clinical implications suggest that, when viewed within the framework presented herein, secondary symptoms can provide important information that can guide clinical diagnosis and treatment.

## Conclusion

The intent of this review is to attract attention to the nonspecific characteristics of psychopathologies and to demonstrate that, within the systems neuroscience framework, these characteristics may hold important information that can advance the theoretical and clinical understanding of psychopathologies. The direct evidence for a relation between behavioral secondary symptoms and network aberrations and vulnerability, as well as its clinical usefulness, is limited and requires substantial research. We hope the integrative framework outlined herein will stimulate work that aims to increase understanding of this relation further and to advance our understanding of the common mechanisms underlying disorders and how they can be used for prevention, diagnosis and treatment of disorders.

## Endnote

^1^Network metrics include measures of local connectivity levels (such as the clustering and modularity of various nodes in the network), measures of global integration (such as the average path length between two nodes, which is indicative of how efficiently the network is connected) and measures of the importance of specific hub nodes (such as the centrality of specific nodes). Another example of a network metric is how much the brain adheres to a global network architecture, called a “small world network,” that reflects high clustering, a short path length and the formation of densely connected hubs [298].

## Abbreviations

AD: Alzheimer disease; ADHD: Attention-deficit/hyperactivity disorder; ASD: Autism spectrum disorder; DTI: Diffusion tensor imaging; EEG: Electroencephalography; fMRI: Functional magnetic resonance imaging; MCI: Mild cognitive impairments; MEG: Magnetoencephalography; NSS: Neurological soft sign; OCD: Obsessive-compulsive disorder; PTSD: Posttraumatic stress disorder.

## Competing interests

The authors declare that they have no competing interests.

## Authors’ contributions

NVL, YG and MD participated equally in the review process and in the manuscript preparation process. All authors read and approved the final manuscript.
